# Color Variations in Left Atrial Appendage Occluders

**DOI:** 10.1016/j.jaccas.2021.10.001

**Published:** 2021-11-17

**Authors:** Ott Saluveer, Nikola Drca, Bo Sahlgren, Mats Jensen-Urstad, Frieder Braunschweig

**Affiliations:** Department of Cardiology, Karolinska University Hospital, Stockholm, Sweden

**Keywords:** atrial fibrillation, echocardiography, left atrial appendage, occluder, stroke, LAA, left atrial appendage, LAAO, left atrial appendage occlusion

## Abstract

The authors stopped a case of left atrial appendage occlusion because of miscolored brownish devices. The investigation demonstrated that devices may show a range of colors from a typical blue to a brownish hue, depending on oxide layer thickness, and this does not appear to have any impact on the performance of the device. (**Level of Difficulty: Intermediate.**)

## Central Illustration

**Figure:**
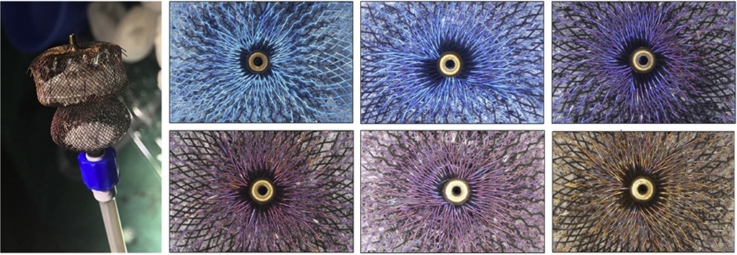

**Figure 1:**
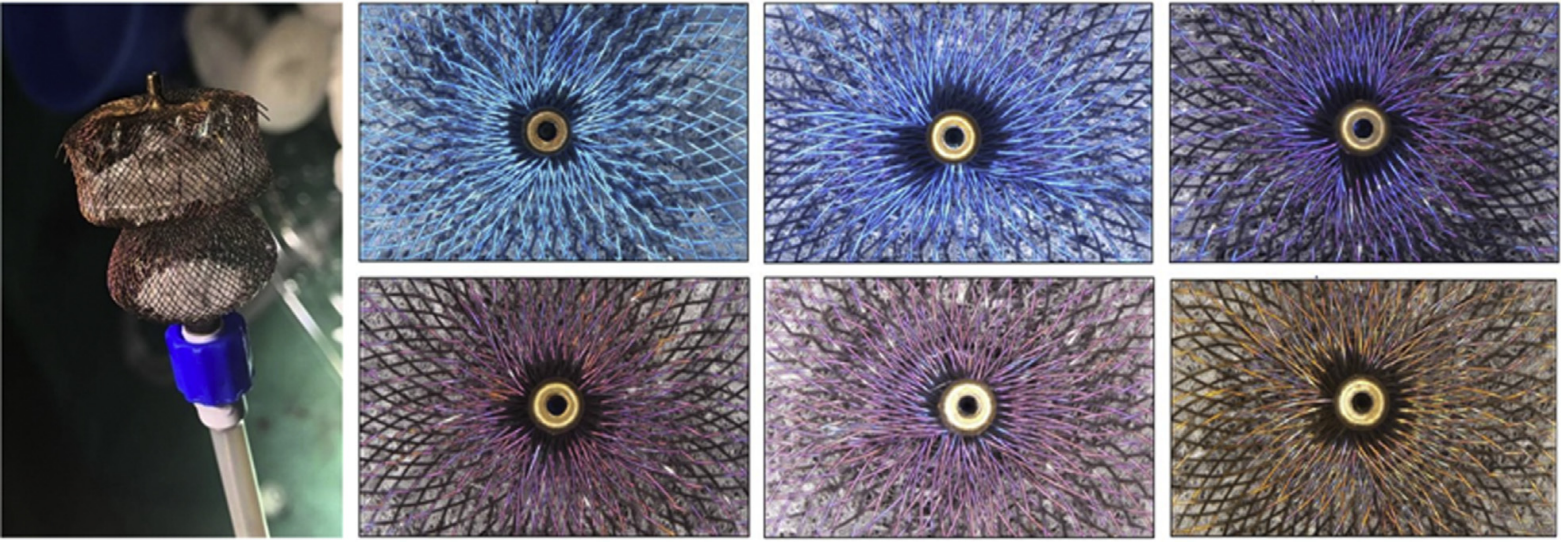
Color Variations in LAA Occluders **(Left)** Left atrial appendage (LAA) occluder from the LAA occlusion (LAAO) procedure showing brownish colors. **(Right)** Original photos of different LAAO devices from the manufacturing line at the Abbott Company showing color variations.

An 81-year old man with paroxysmal atrial fibrillation was referred to our hospital for percutaneous left atrial appendage occlusion (LAAO). The LAAO procedure was performed with patient under general anesthesia. Based on left atrial appendage (LAA) measurements using computed tomography, transesophageal echocardiography, and LAA angiography, a 28-mm LAA occluder device (Amplatzer Amulet Occluder, Abbott Structural Heart) was chosen and placed in the LAA through a 14-F delivery sheath. The stability criteria were not fully met, and we downsized to a 25-mm device instead. Preparing the device, we observed that the color was not, as expected, a blue hue, but instead the device showed brownish colors (Figure 1). A decision was made not to use this device because of uncertainties about the cause of the unexpected color and its potential impact on the performance of the device. Four different 25-mm device boxes were opened, with different degrees of brownish color on the devices. The procedure was stopped and rescheduled.

## Testing and Inspections

The four devices were returned to the manufacturer for investigation. No deficiencies were found related to materials, batches, or process that would implicate a potential impact on the performance of the devices. The devices were inspected on both the macroscopic and the microscopic level. All four devices were loaded into their loaders and were flushed with water. The water ran clear in all instances, and no material or color was observed in the water. On the microscopic level, the devices were inspected by stereoscopy, scanning electron microscopy, and energy-dispersive spectrometry. No evidence of corrosion was found on any device.

## Why Do Devices Exhibit Different Colors?

The occluders contain wires of nitinol, a nickel-titanium alloy. Nitinol forms a thin oxide layer on the surface, and the color variation range is associated with the oxide layer thickness that develops during thermal setting in the manufacturing process. The oxide layer is very thin (80-100 nm), consisting primarily of titanium oxide, and is made consistent using a chemical etching process. The nitinol wire is approximately 300 to 2,000 times greater than the oxide layer, and the oxide layer does not affect the mechanical properties of the wire. Different oxidation states in combination with variations in the oxide layer thickness result in different colors, from a blue to a brownish hue, caused by a light interaction with the oxide layer (Figure 1).

## Conclusions

The present findings show that color variations in LAA occluders may occur as a result of the variable nature of the oxidation process during the manufacturing process, and this does not appear to have any impact on the performance of the device.

## Funding Support and Author Disclosures

Dr Sahlgren is a proctor for Abbott and has received consultant fees. All other authors have reported that they have no relationships relevant to the contents of this paper to disclose.

